# Non-Targeted Screening and Quantitative Analysis of Pesticides and Veterinary Drug Residues in *Brassica rapa chinensis* Using an Improved Quechers Method Based on Magnetic Materials

**DOI:** 10.3390/foods14193288

**Published:** 2025-09-23

**Authors:** Minmin Tang, Yongbiao Ni, Tianli Zang, Wei Gao, Jinzhu Song, Jie Zou, Danke Xu

**Affiliations:** 1 Key Laboratory of Food Contact Materials Safety, State Administration for Market Regulation, Jiangsu Product Quality Testing & Inspection Institute, Nanjing 210007, China; 2 State Key Laboratory of Analytical Chemistry for Life Science, School of Chemistry and Chemical Engineering, Nanjing University, Nanjing 210023, China

**Keywords:** QuEChERS, magnetic MWCNTs, magnetic PSA, non-targeted analysis, orbitrap, *Brassica rapa chinensis*

## Abstract

An improved QuEChERS method based on magnetic nanomaterials was established, and coupled with ultra-high-performance liquid chromatography and a quadrupole electrostatic field orbitrap mass spectrometer, enabling non-targeted screening and quantitative analysis of commonly used pesticide and veterinary drug residues in *Brassica rapa chinensis*. The combination of magnetic PSA and magnetic multi-walled carbon nanotubes (MWCNTs) was utilized for purification of 121 organic compounds spanning a wide range of polarities. Spiking experiments showed that the developed non-target screening workflow exhibited convincing identification results, with over 89.3% of chemical substances being screened out even at spiking levels as low as 2 μg/kg. The linear ranges of these target substances were between 1 and 200 μg/kg, with determination coefficients all exceeding 0.99. The limits of quantification were between 2 μg/kg and 10 μg/kg. The average recovery rates were in the range of 61.7% to 121.6%. Nineteen compounds were confirmed (meeting all the restriction conditions) and quantified from *Brassica rapa chinensis* collected from vegetable-planting bases in Jiangsu Province. The results indicated that the magnetic bead-based pretreatment strategy can be utilized for the simultaneous screening and quantitative analysis of pesticide and veterinary drug residues in *Brassica rapa chinensis*.

## 1. Introduction

The safety of agricultural products is the foundation of food safety, which is related to the health of the general public and the sustainable development of agriculture and rural economy. Pesticides and veterinary drugs are important agricultural inputs. Pesticides can prevent and control diseases and pests, ensure increased crop yields, while veterinary drugs can prevent and control livestock and poultry diseases and increase animal body mass and feed conversion rate. However, a portion of pesticides adheres to crops, while another portion is scattered in environments such as soil, atmosphere, and water. Some of the residual pesticides in the environment are absorbed by plants. On the other hand, 30–90% of the veterinary drugs are excreted from the animal’s body in the form of raw materials or metabolites [1]. With the use of organic fertilizers, animal manure as a green organic fertilizer can lead to veterinary drugs entering the soil environment, resulting in pesticide and veterinary drugs pollution in farmland soil [2]. These organic pollutants will also migrate and accumulate in the soil–vegetable system. Finally, the vegetables will be transmitted through the food chain, seriously threatening human health and environmental safety.

Most individual target methods have been used for detecting pesticide or veterinary drug residues by liquid chromatography tandem mass spectrometry (LC-MS/MS) or gas chromatography tandem mass spectrometry (GC-MS/MS), which can achieve high sensitivity and accuracy in quantitative analysis of known substances [3,4,5,6]. However, due to the limitations of scanning speed and dwell time in triple-quadrupole mass spectrometry, the detection of hundreds of targets often requires grouping and establishing multiple detection methods, and it may miss out on unknown but predominant pollutants. Moreover, achieving qualitative detection is susceptible to interference in complex sample matrices. High-resolution mass spectrometry (HRMS) technology such as quadrupole time-of-flight (QTOF) or orbitrap has the advantages of high mass accuracy, high resolution, and a fast scanning speed [7], making it possible to detect and confirm unknown pollutants in the environment and in food that pose a threat to human health. However, due to differences in instrument interfaces, ionization, applied voltage, and mobile phase composition, etc., uncertainty is introduced in screening analysis, which also leads to insufficient screening accuracy of commercial mass spectrometry libraries and limits their application [8]. To our knowledge, due to the significant differences in the properties of pesticides and veterinary drugs, most of the reported research focused on separate detection of veterinary drugs and pesticides [9,10,11]. Chen et al. used HRMS fingerprinting to explore micropollutant contamination in soil and vegetables caused by swine wastewater irrigation, but only focused on the identification of pollutants and did not pay attention to detection [12]. Therefore, it is crucial to establish an effective method for rapid screening and quantitative analysis of potential pesticides and veterinary drugs in vegetables.

At present, many sample preparation techniques have been used for the extraction and purification of pesticide residues in vegetables mainly including solid-phase extraction (SPE) and QuEChERS (Quick, Easy, Cheap, Effective, Rugged, Safe) pretreatment techniques. However, a simple and universal method for simultaneously detecting multiple veterinary drugs and pesticides residues in vegetables has yet to be established, since the sample preparation of veterinary drugs and pesticides is usually different. SPE can achieve good enrichment and purification, but its limitation is that it can only enrich a limited number of target compounds with similar properties due to the enrichment adsorbents. The QuEChERS method originated from the analysis of pesticide residues in vegetables and fruits, which can effectively shorten the procedure and reduce manual labor and the use of organic reagents [13], and was subsequently promoted to other substrates and target substance detection. It is based on the principle of purification rather than enrichment. The core of the QuEChERS method is different adsorbents, commonly including ethylenediamine-N-propylsilane silica gel (PSA), octadecyl (C18), graphitized carbon black (GCB) [14,15]. The conventional QuEChERS pretreatment method coupled with LC-HRMS has been successfully validated and applied to the non-target screening of organic chemicals [16,17]. Recently, multi-walled carbon nanotubes (MWCNTs) have exhibited strong adsorption capacity due to their graphene layers [18]. The current consensus is that compared to GCB, MWCNTs can reduce the adsorption of certain planar structure substances. However, the separation between the adsorbents and solution require the use of centrifuges, significantly diminishing the efficiency of pretreatment. Magnetic nanoparticles have high enrichment ability and extraction efficiency due to their nanoscale particle size, large specific surface area, and short diffusion path. Furthermore, the separation process can be facilitated through the use of a magnet. Iron oxide (Fe_3_O_4_) is commonly employed due to its low cost, low toxicity, and ease of synthesis and modification, demonstrating strong application potential [19].

In this study, a simple, fast, and effective magnetic QuEChERS clean-up procedure for simultaneous determination of pesticide and veterinary drug residues in *Brassica rapa chinensis* has been developed. The magnetic MWCNTs were synthesized as purification materials, and the effectiveness of different magnetic materials in sample purification was compared. To evaluate the method performance, 121 compounds, including 63 representative pesticides and 58 veterinary drugs, were selected as quality controls to verify the reliability of screening and quantification by UHPLC Orbitrap MS. The recovery, matrix effect, as well as method limits of detection were validated. To our knowledge, this study is the first to establish a magnetic QuEChERS method combined with HRMS for simultaneous analysis of veterinary drugs and pesticides in *Brassica rapa chinensis*. The proposed method not only serves as a useful tool for routine monitoring, but also provides technical support for agricultural product safety risk assessment and control.

## 2. Materials and Methods

### 2.1. Chemicals, Reagents, and Instruments

Acetonitrile (ACN), methanol (MeOH) (LC-MS grade) were purchased from Merck (Darmstadt, Germany). Ammonium formate, formic acid (FA) (HPLC grade), anhydrous sodium sulfate, magnesium sulfate, sodium chloride, (NH_4_)_2_Fe(SO_4_)_2_·6H_2_O, FeCl_3_·6H_2_O and FeCl_2_·4H_2_O (AR), and ammonia (25% *w*/*w*) were provided by Aladdin Co. (Shanghai, China). MWCNTs (20–30 nm in diameter, 10–30 μm in length) and Fe_3_O_4_-GO (10–30 nm in diameter) were purchased from Nanjing Xianfeng Nanotechnology Co., Ltd (Nanjing, China). Fe_3_O_4_-SiO_2_-PSA (300–400 nm) and Fe_3_O_4_-SiO_2_-C_18_ (300–400 nm) were purchased from PuriMag Biotechnology Co., Ltd. (Shenzhen, China).

The stock solutions of 63 representative pesticides and 58 veterinary drugs standards (100 μg/mL, purity > 98.5%) were purchased from BePure Biotechnology Co., Ltd. (Shanghai, China). All the standards used were listed in Table S1. The mixed standard solutions of pesticides and veterinary drugs were diluted in MeOH at 1 μg/mL and maintained at −18 °C in glass vials.

An Orbitrap Exploris 120 mass spectrometer coupled to a Thermo Scientific Vanquish ultra-high-performance liquid chromatography (Thermo Fisher Scientific, Waltham, MA, USA) was used for analysis. Transmission electron microscopy (TEM) was applied on the nanomaterial using a FEI Tecnai F20 instrument (FEI, Hillsboro, OR, USA).

### 2.2. Synthesis of Magnetic Multi-Walled Carbon Nanotubes (MG-MWCNTs)

The synthesis method has been modified based on the literature [20]. Firstly, 0.85 g (NH_4_)_2_Fe(SO_4_)_2_·6H_2_O and 0.42 g FeCl_3_·6H_2_O were weighed and dissolved in 250 mL of water, then 0.5 g MWCNTs was added and the mixture was stirred to form a suspension. The suspension was treated with ultrasound at 50 °C for 20 min. During this process, 25 mL of 8 mol/L ammonia solution was added dropwise using a constant-flow pump to precipitate Fe_3_O_4_ on the MWCNT wall, and the mixture was kept at 50 °C for 30 min. Subsequently, the magnetic nanocomposite materials (MG-MWCNTs) were cooled to room temperature and washed three times with pure water and ethanol. Finally, the collected MG-MWCNTs were vacuum-dried overnight at 60 °C and stored for later use.

### 2.3. Sample Preparation

The *Brassica rapa chinensis* samples were obtained from the local supermarket in Nanjing, China. First, 2 kg of *Brassica rapa chinensis* was chopped after the removal of surface dust and foreign matter, thoroughly mixed, and a portion was homogenized using the quartering method for later use [21]. The samples were experimentally verified to be free of the target compounds. Next, 10 g of homogenized samples was weighed in a 50 mL plastic centrifuge tube. Then, 2 mL of EDTA-McIlvaine buffer (0.1 mol/L, pH = 4.0) and 8 mL of ACN containing 1% FA were added in order to extract the targets. The mixture was vortexed for 1 min and sonicated in an ice bath for 10 min and subsequently added with 4 g of MgSO_4_ and 1 g of NaCl by shaking for another 5 min. After centrifugation for 5 min at 5000 r/min, 2 mL of supernatant was taken and added to a centrifuge tube pre-filled with 5 mg MG-MWCNTs and 15 mg Fe_3_O_4_-SiO_2_-PSA. The mixture was vortexed in a centrifuge tube for 1 min, then magnetic separation was quickly performed using a magnet. The supernatant was transferred and filtered through a 0.22 μm microporous membrane for testing.

### 2.4. Determination of Co-Extracts and ME

The purification effects were evaluated by comparing the removal rates of co-extracted substances in the sample matrix before and after magnetic bead purification. The amounts of co-extracted substances were determined by the gravimetric method [22]. The details of the process are presented in supporting information. The matrix effect (ME) can be calculated according to the following formula: ME (%) = B/A × 100. Among them, A is defined as the peak area of a pure standard solution of a certain concentration, and B is defined as the peak area of a matrix matching a standard solution of the same concentration [23].

### 2.5. Instrument Conditions

The separation was performed on a Waters ACQUITY UPLC BEH C18 chromatography column (100 mm × 2.1 mm, 1.7 μm) at a column oven temperature of 40 °C. The mobile phase involved 2.5 mmol/L ammonium formate containing 0.1% FA in water (mobile phase A) and MeOH (mobile phase B). The gradient elution program was as follows: 0.5 min 2% B, 1.0 min 50% B, 15.0 min 95% B, 17.0 min 95% B, and 17.1 min 2% B. The flow rate was 0.3 mL/min, and the injection volume was 2 μL.

The positive and negative ion detection modes (ESI^+^, ESI^−^) were scanned simultaneously; this was performed by OE-120 with HESI ionization. The spray-positive voltage was 3.5 kV, while the spray-negative voltage was 2.5 kV. The ion transfer tube temperature was 320 °C. The evaporator temperature was 350 °C. The sheath gas was 40 arb, the auxiliary gas was 10 arb, and the sweep gas was 1 arb. Fast screening and quantitative analysis were acquired using the Full MS data-dependent MS_2_ (dd MS_2_) mode full-scan mass range from 100 to 900 *m*/*z*. Through the full scan and automatic triggering of secondary mass spectrometry data-dependent scanning, precise molecular weights of precursor ions and product ions were obtained within the set scanning ranges. The resolution was 120,000 full width at half maximum (FWHM) and a dd MS_2_ resolution of 30,000 FWHM. The automatic gain control (AGC) of the full-scan MS was set to standard, with a maximum injection time of 100 ms. The filter parameters were set to ensure that the intensity of the primary ion peak exceeded 5 × 10^4^ and the signal-to-noise ratio was higher than 50, and the threshold intensity of the product ion peak was 1 × 10^3^. Prior to data collection, a positive and negative ion calibration solution was used for mass axis calibration.

The compound identification and confirmation were based on a self-built library containing the collision induced-dissociation of all product ions, and an accurate mass of 602 pesticides and 88 veterinary drugs developed by our research group. Automatic analyte identification and confirmation were based on HRAM measurements of the precursor and its characteristic product ions, with a mass error of ±5 ppm, a retention time tolerance of ±0.1 min, and product ions with a mass error of ±5 ppm. An isotope matching score higher than 75 was considered as an additional filter. Xcalibur 4.4 and Trace Finder 5.1 software (Thermo Scientific, Waltham, MA, USA) were used for instrument control and data acquisition and processing.

### 2.6. Method Validation

To evaluate the efficiency of magnetic QuEChERS and non-targeted methods, method validation was conducted on 63 representative pesticides and 58 veterinary drugs. The validation considered linearity, limits of detection, limits of quantification, recovery rate, and matrix effects [24]. The quantitative method was established using the Trace Finder software. In terms of linear range, eight calibration samples (1, 2, 5, 10, 20, 50, 100, and 200 μg/kg) were analyzed to obtain satisfactory calibration curves. The matrix-matching standard curves were plotted with the peak area of the mass spectrometry primary peak as the y-axis and concentration as the x-axis. The selection of quantitative limits (LOQs) not only met the requirements for target confirmation in Section 2.5, but also needed to meet the requirements for the recovery rate and accuracy (RSD ≤ 20%). The recovery rate tests with three repeated spiked tests conducted at three levels were used to investigate the accuracy and precision. Generally speaking, for quantitative methods, the recovery rate range needed to reach 60–120% [25].

## 3. Results and Discussion

### 3.1. Characterization of MG-MWCNTs

**Figure 1:**
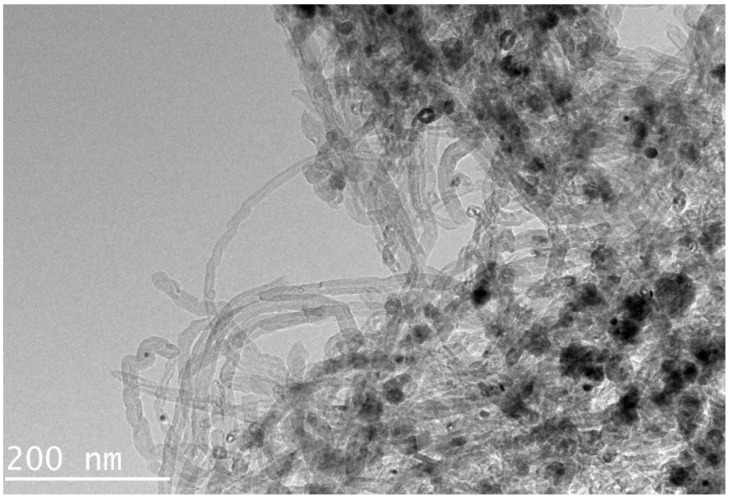
TEM images of MG-MWCNTs.

The morphology of MG-MWCNTs composite is characterized by TEM in Figure 1. The images clearly show that MG-MWCNTs are tubular, embedded with spherical Fe_3_O_4_ nanoparticles. Only a few negligible bonding sites are occupied during this synthesis process. Therefore, the adsorption properties of MWCNTs remain unchanged [26].

### 3.2. Optimization of Pre-Processing

#### 3.2.1. Optimization of Extraction Conditions

The selection of extraction conditions is mainly related to the physical and chemical properties of the target substances, as well as the properties of the extract solvent. In this study, the extraction conditions are a necessary prerequisite for ensuring the performance of the method due to the wide applicability of extracting targets with different properties. According to some reports [27,28], acidic ACN has a good protective effect on pesticides with poor stability. In veterinary drugs extraction, EDTA-McIlvaine buffer can prevent tetracycline drugs from forming insoluble salts with Ca^2+^ and Mg^2+^ in the matrix, which can improve extraction efficiency. Therefore, the effects of ACN (1% FA), ACN (1% AcH), ACN, and ACN (1% FA): EDTA = 4:1 on the extraction efficiency of 121 target compounds were first compared.

**Figure 2:**
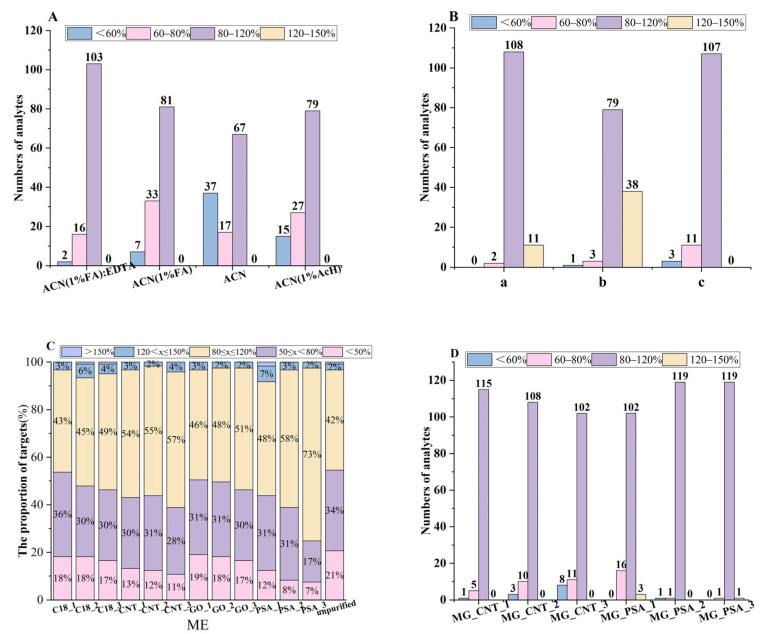
The effect of pre-processing on the recovery and ME distribution of the targeted compounds: (**A**) solvent extraction on recovery; (**B**) salt types on recovery; (**C**) magnetic nanomaterials on ME; (**D**) magnetic nanomaterials on recovery (1 represented 5 mg, 2 represented 10 mg, and 3 represented 15 mg).

As shown in Figure 2A, when using acidic ACN for extraction, at least 106 targets (more than 85%) had a recovery rate ranging from 60% to 120%, which was significantly higher than 84 targets (69.4%) in ACN alone. This was mainly because acidic ACN has a significantly better extraction effect on veterinary drugs than ACN alone, and ACN (1% FA) had a better extraction effect on fluoroquinolone drugs than ACN (1% AcH). The results also showed that after, when ACN (1% FA):EDTA = 4:1, the recovery rates of 103 targets (85.1%) ranged from 80% to 120%, which was the highest result. Therefore, ACN (1% FA):EDTA = 4:1 was selected as the type of extraction solution to be used for subsequent extractions.

Combination with salt is the core step of the QuEChERS method, which can help phase separation and remove water from the sample, directly affecting the extraction efficiency and subsequent purification effect. In order to investigate the effect of salt types on extraction efficiency, the experimental conditions were set as follows: (a) 4 g anhydrous MgSO_4_ and 1 g NaCl; (b) 4 g anhydrous Na_2_SO_4_ and 1 g NaCl; (c) 4 g anhydrous MgSO_4_, 1 g NaCl, 1 g sodium citrate, and 0.5 g disodium citrate. The results in Figure 2B show that in the MgSO_4_ system, 108 targets (89.3%) obtained a recovery rate ranging from 80% to 120%, and the effect was significantly better than that in the Na_2_SO_4_ system. The buffer system of sodium citrate had no significant effect on the recovery rates of the target substance, but instead affected the extraction efficiency of tetracycline substances. MgSO_4_ has the ability to quickly absorb and extract water from solvents, and its heating process can significantly improve liquid–liquid distribution efficiency [29]. NaCl can affect the polarity of the extraction solvent, thereby improving the extraction selectivity [30]. Therefore, 4 g of anhydrous MgSO_4_ and 1 g of NaCl were selected for subsequent experiments.

#### 3.2.2. Optimization of Magnetic Nanomaterials

Firstly, four types of magnetic nanomaterials, including MG-PSA, MG-GO, MG-C_18_, and synthesized multi-walled carbon nanotube MG-MWCNTs that are capable of removing organic acids and pigments from vegetables, were selected as purification materials. The purification effects of different magnetic nanomaterials in amounts of 5 mg, 10 mg, and 15 mg were evaluated based on ME and co-extracts removal rates. The ME values are crucial for obtaining reliable results in MS analysis. ME can be divided into three situations. A range of 80–120% represents negligible enhancement; a range of 120–150% or 50–80% represents moderate enhancement or inhibition; a range higher than 150% or less than 50% represents strong enhancement or inhibition [31].

The ME results in Figure 2C show that without purification with magnetic nanomaterials, only 43% of the target substances had ME values within the range of 80–120%. MG-C_18_ mainly removed non-polar substances (such as fats) through non-polar interactions, so its effect was not significant in vegetables with low oil content. After purification with four different magnetic beads, it was found that MG-GO was not as good as MG-MWCNTs and MG-PSA had the most significant improvement effect on ME. Moreover, the improvement effect of ME increased with the increase in the amounts of magnetic nanomaterials. When the amounts of MG-PSA and MG-MWCNTs reached 15 mg, the proportions of target substances with ME values ranging from 80% to 120% were 73% and 57%, respectively.

The purification efficiency of four magnetic nanomaterials was further evaluated by the removal rates of the co-extracts. As shown in Table 1, the results demonstrated that MG-PSA and MG-MWCNTs have the highest removal efficiency for co-extracts, which can also be mutually confirmed by the ME results.

In addition to considering ME, purification materials should not affect the recovery of target substances. Therefore, we investigated the adsorption of 121 target substances by MG-MWCNTs and MG-PSA at different dosages. As shown in Figure 2D, with the increase in MG-MWCNTs amounts, the numbers of the target substances decreased from 115 (95%) to 102 (84%) within the range of 80–120%. When the MG-MWCNTs amount exceeded 10 mg, it had a significant adsorption effect on several pesticides such as carbendazim, chlorfenapyr, diflubenzuron, and chlorpyramide, as well as on fluoroquinolone veterinary drugs. With the increase in MG-PSA usage, the recovery rates of most substances improved, which may be attributed to the removal of acidic interferents from vegetables. Therefore, a combination of 5 mg MG-MWCNTs and 15 mg MG-PSA was chosen as the magnetic nanomaterial to be used for purification. Subsequently, the impact of the combination on the recovery rates of 121 target substances was verified, and it was found that the recovery rates of 108 target substances ranged from 80% to 120%.

The commonly used purification materials in the conventional QuEChERS method include PSA, C_18_, and graphitized carbon black (GCB). GCB is a carbon material with a multi-ring planar structure that can adsorb substances such as pigments and sterols in samples, but it is also prone to adsorbing compounds with planar structures. MG-MWCNTs are porous graphite cylindrical nanomaterials with a large specific surface area, strong adsorption capacity, good chemical stability, easy preparation, and low cost. They can adsorb impurities such as pigments, phenols, sterols, and organic acids in a sample. Figure S1 shows that compared with the extraction solution without magnetic materials purification, the solution obtained by combining 15 mg MG-PSA and 5 mg MG-MWCNTs has a lighter color. Moreover, we also compared the colors under a combination of 50 mg PSA and 5 mg GCB adsorbents in the conventional QuEChERS, proving that the mixture of MG-PSA and MG-MWCNTs had a stronger ability to adsorb pigments.

### 3.3. Qualitative Analysis

Mass spectrometry libraries are the foundation for quickly screening unknown pollutants. Compared with the commercial database provided by the instrument manufacturers, the self-built library contains more comprehensive information on secondary fragment ions and collision energy, resulting in higher accuracy in compound matching. To verify the applicability of the self-built spectral library in real detection, the spiked samples were used to validate the performance of the screening method using the optimized processing procedures. The matching results were based on five indicators, including the mass-to-charge ratio (*m*/*z*), retention time (RT), fragment ions (FI), isotopic pattern (IP), and library search (LS). The software provided the deviation between the measured and theoretical values of each retrieval parameter, and determined the screening results based on the degree of matching. In qualitative screening, the parameters included *m*/*z* within a mass error limit of ±5 ppm and RT within ±0.1 min. In order to determine the screening detection limit (SDL), a total of 121 target compounds were spiked at 0.5, 1, 2, 5, and 10 μg/kg in *Brassica rapa chinensis* samples and analyzed in replicates (*n* = 20).

**Figure 3:**
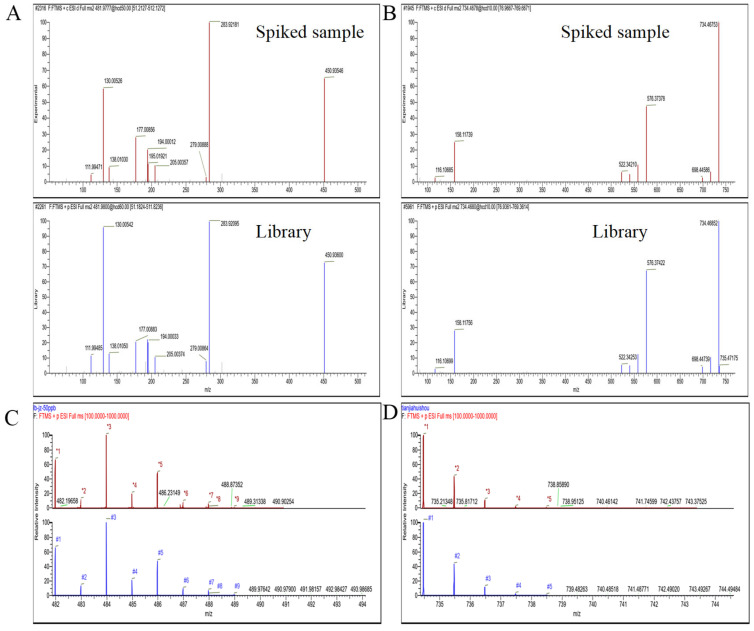
MS/MS fragment ion matching of the spiked sample and library (**A**,**B**) and isotopes (**C**,**D**) of chlorantraniliprole and erythromycin.

The matching results can also be directly displayed through Trace Finder software. The SDL of 1 μg/kg and 2 μg/kg was applicable to 87 targets (71.9%) and 108 targets (89.3%) in *Brassica rapa chinensis*, respectively, based on the screening results (precursor ion with 5 ppm of mass error and RT threshold of ±0.1 min). The remaining compounds were detected at the SDL of 5 μg/kg. By setting the matching mode to reverse matching, it is possible to determine whether the target substance is present in the sample. Taking the potential pesticide and veterinary drug residual chlorantraniliprole and erythromycin as examples, as shown in Figure 3, the results showed that the two spiked substances in *Brassica rapa chinensis* matched well with the fragment ion information of MS/MS in the database. Nine out of nine chlorantraniliprole isotope numbers were successfully matched, and for erythromycin, 5 of 5 isotope numbers were matched successfully, both achieving matching scores of 100. This indicated that the method can quickly screen for various potential residual pesticides and veterinary drugs in *Brassica rapa chinensis*.

### 3.4. Quantitative Analysis

#### 3.4.1. Matrix Effect (ME)

**Figure 4:**
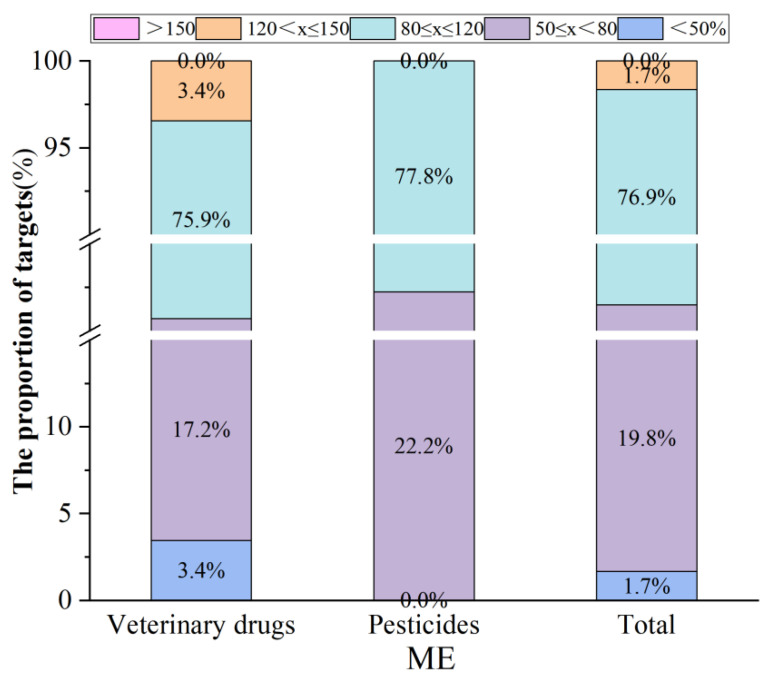
The ME of pesticides and veterinary drugs in the established magnetic QuEChERS method.

ME is caused by matrix substances in the sample, which not only inhibit or enhance the detection signal of the target substance, but also easily affect the accuracy of detection. The optimized method was used to verify the ME on pesticide and veterinary drug targets. As shown in Figure 4, the proportion of target substances with negligible matrix effects in the range of 80–120% was 76.9%, and the proportion of target substances with moderate inhibition or enhancement was 21.5%. From the perspective of target types, the purification effect of magnetic QuEChERS was more advantageous for pesticides. Compared with using individual types of magnetic nanomaterials, the number of target substances with strong or inhibitory properties was significantly reduced, with only two veterinary drugs in the strong inhibitory range.

#### 3.4.2. Linear Range, Quantification Limit, and Recovery Rates

The matrix-matching standard curves with series concentrations of 1, 2, 5, 10, 20, 50, 100, and 200 μg/kg were prepared to evaluate linear relationships. The standard curves were plotted based on the peak area (y) and concentration (x) of each component. The optimal concentration range for each compound was selected based on different response strengths, and each target compound showed a good linear relationship within an appropriate linear range, with a coefficient of determination (R^2^) greater than 0.98. More than 60% of the target compounds had R^2^ values exceeding 0.999, which was beneficial for accurate quantification (Table 2).

In addition to meeting the confirmation requirements, the LOQs also needed to meet the requirements of recovery rate and precision. Overall, the LOQs of 121 targets were from 2 to 10 μg/kg. This was superior to the quantitative limit requirements of National Food Safety Standards such as GB 23200.121-2021 [21] and EN 15662: 2018 [32], and was sufficient to analyze the target compounds at concentrations below the maximum residue limits. The accuracy and precision of the established method were evaluated by recovery rates and relative standard deviations (RSDs) at three concentration levels. At the spiking levels of 1, 2, and 20 times their respective LOQs, the average recovery rates of each compound ranged from 61.7% to 121.6%, and the RSD ranged from 0.1% to 19.9% (as shown in Table 2), indicating that this magnetic QuEChERS method had good accuracy and repeatability. For pesticides, the recovery rates of 96.8% pesticides ranged from 80% to 120%. The target substances with a recovery rate around 70% were mainly some of fluoroquinolone drugs (enoxacin, norfloxacin, sarafloxacin) and two tetracycline drugs (doxycycline and oxytetracycline) in veterinary drugs. Tetracyclines are mainly limited by the efficiency of the extraction solution. As for fluoroquinolones, there is a π-π interaction between the surface of MG-MWCNTs and the benzene ring in fluoroquinolones, resulting in the loss of some drugs [33].

**Figure 5:**
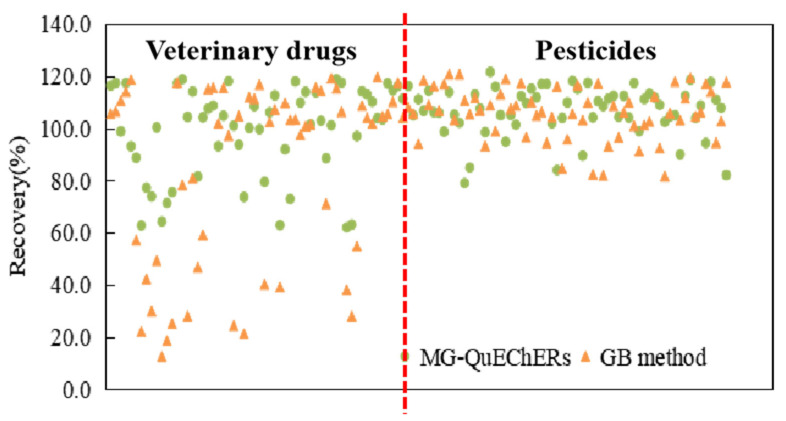
Comparison of pesticides and veterinary drugs recovery rates in *Brassica rapa chinensis* using two types of QuEChERS method.

At present, there is a lack of research and standardized methods for detecting veterinary drug residues in plant-based products. The established magnetic QuEChERS method and the GB 23200.121-2021 QuEChERS method are compared in Figure 5, which depicts the recovery rates of the target substances. Both methods can achieve good recovery rates for pesticides, with the recovery rates for 61 pesticides (accounting for 96.8%) ranging from 80% to 120%. However, it was found that GB 23200.121-2021 had a weak ability for veterinary drug detection, and the recovery rates of some veterinary drug compounds were less than 60%. This was largely attributed to the selection and dosage of the extraction liquid type and purification material. In addition, compared to traditional dSPE purification materials, the synthesis and operation of magnetic materials are simple, and they have stronger purification effects, which are not limited to laboratory centrifuge equipment.

### 3.5. The Detection of Real Samples

Qualitative and quantitative testing were conducted on 121 target compounds in 50 batches of vegetables from planting bases in Jiangsu Province. The results in Table 3 show that 33 out of the 50 batches of samples have been screened for target compounds, including 21 pesticides and 2 veterinary drug residues. The compound with the highest detection frequency was dimethomorph, with an average content range of 2.13–1184 µg/kg. According to the National Food Safety Standard for Maximum Residue Limits (GB 2763-2021) [34].of Pesticides in Food, a batch of *Brassica rapa chinensis* samples with chlorpyrifos content exceeding the limit requirement by 0.02 mg/kg are considered unqualified products.

In addition, we also detected two types of veterinary drug residues, namely doxycycline and ofloxacin in 50 batches of *Brassica rapa chinensis*, with concentrations of 6.40–8.29 µg/kg and 3.1 µg/kg, but they did not exceed the LOQs of the method. This was mainly due to the migration of antibiotic residues from the soil to vegetables. In animal-derived foods, ofloxacin is not permitted, and tetracycline drugs should not exceed 100 µg/kg in animal muscles. However, there is no relevant regulation on the maximum residue limit of veterinary drugs in *Brassica rapa chinensis* and other plant products. From the perspective of daily intake, the intake of vegetables is usually higher than that of meat, so the total exposure of vegetables may be higher. Therefore, the residues of veterinary drugs in vegetables should also be of concern.

## 4. Conclusions

An improved QuEChERS method using magnetic PSA and magnetic MWCNT coupled with UPLC Q-Orbitrap has been developed for simultaneous determination of pesticides and veterinary drug residues in *Brassica rapa chinensis*. In this study, 63 pesticides and 58 veterinary drugs with broad polarity were used as quality control compounds. Combined with self-built HRMS libraries, high-throughput and rapid screening without a standards reference has been achieved. In an analysis of 50 batches of *Brassica rapa chinensis* samples from the planting base, 21 pesticides and 2 veterinary drugs were detected (veterinary drugs did not exceed LOQs). This method is accurate and reproducible, and the key is the use of magnetic materials as adsorbents, which is simple and efficient, greatly simplifying sample pretreatment. It can be used for rapid screening and confirmation of various pesticides and veterinary drug residues in Brassca rapa chinensis, providing technical basis for vegetable regulatory analysis and quality supervision.

## Figures and Tables

**Table 1:** Effects of different nanomaterials on the removal efficiency of co-extracts (*n* = 3).

Types of Magnetic Nanomaterials	MG-PSA			MG-C_18_			MG-GO			MG-MWCNTs			Unpurified *
	5 mg	10 mg	15 mg	5 mg	10 mg	15 mg	5 mg	10 mg	15 mg	5 mg	10 mg	15 mg	
Weight of co-extracts (mg)	5.8	5.7	4.9	5.9	5.7	5.6	5.6	5.4	5.3	5.1	4.8	4.4	5.9
Removal rate (%)	1.7	3.4	16.9	0.0	3.4	5.1	5.1	8.5	10.2	13.6	18.6	25.4	/

* Unpurified means the solution has not been purified by any magnetic nanomaterials.

**Table 2:** The quantitation limits, recoveries, precision, calibration curve and determination coefficient of the method.

NO.	Compounds	Linear Range μg/kg	Calibration Curve	R^2^	SDL μg/kg	LOQ μg/kg	Spiking Level (*n* = 3)		
							LOQ	2 × LOQ	20 × LOQ
1	Atrazine	1–50	y = 5.56 × 10^6^x + 3.89 × 10^5^	0.9999	1	2	117.6 (9.7)	118.5 (4.4)	114.4 (4.5)
2	Azoxystrobin	1–50	y = 6.39 × 10^6^x − 4.13 × 10^6^	0.9996	1	2	88.8 (10.2)	95.9 (6.6)	106.1 (5.9)
3	Betamethasone	1–50	y = 5.06 × 10^5^x + 4.56 × 10^4^	0.9998	1	2	87.8 (3.5)	104.1 (9.6)	108.4 (1.5)
4	Boscalid	1–50	y = 1.41 × 10^6^x − 1.02 × 10^6^	0.9999	1	2	119.1 (2.6)	109.8 (10.9)	98.7 (1.8)
5	Carbendazim	1–50	y = 4.31 × 10^6^x − 1.64 × 10^6^	0.9985	1	2	102.0 (3.6)	105.4 (2.3)	113.9 (5.0)
6	Carbofuran	1–50	y = 3.53 × 10^6^x + 8.14 × 10^6^	0.9993	1	2	72.2 (13.3)	105.0 (5.0)	105.2 (1.5)
7	Chlorantraniliprole	1–50	y = 5.62 × 10^5^x − 5.81 × 10^5^	0.9995	1	2	103.5 (19.5)	98.2 (15.8)	102.1 (2.5)
8	Chlorpyrifos	1–50	y = 1.53 × 10^6^x − 4.25 × 10^5^	0.9998	1	2	78.8 (7.9)	94.5 (4.6)	113.1 (3.5)
9	Clarithromycin	1–50	y = 2.84 × 10^5^x − 4.30 × 10^4^	1.0000	1	2	118.2 (3.2)	109.3 (4.7)	93.8 (2.1)
10	Dexamethasone	1–50	y = 5.06 × 10^5^x + 4.57 × 10^4^	0.9998	1	2	88.0 (1.3)	101.9 (10.4)	106.2 (1.4)
11	Dichlorvos	1–50	y = 1.27 × 10^6^x − 2.69 × 10^5^	1.0000	1	2	114.5 (19.6)	117.1 (0.8)	116.0 (4.5)
12	Difenoconazole	1–50	y = 1.47 × 10^6^x − 8.57 × 10^5^	0.9998	1	2	117.0 (16.7)	115.3 (8.6)	105.1 (4.1)
13	Diflubenzuron	1–50	y = 6.56 × 10^5^x − 2.80 × 10^5^	0.9993	1	2	97.2 (9.6)	83.3 (6.7)	94.8 (0.3)
14	Dimethomorph	1–50	y = 1.28 × 10^6^x − 4.19 × 10^4^	0.9992	1	2	109.6 (9.8)	118.4 (0.9)	116.3 (4.7)
15	Etofenprox	1–50	y = 2.84 × 10^6^x − 3.18 × 10^6^	0.9996	1	2	112.1 (9.5)	108.1 (0.6)	101.4 (4.2)
16	Fipronil	1–50	y = 1.57 × 10^6^x + 1.05 × 10^5^	0.9997	1	2	107.3 (6.2)	97.7 (0.9)	115.2 (2.3)
17	Fipronil desulfinyl	1–50	y = 1.78 × 10^6^x + 1.17 × 10^4^	0.9993	1	2	105.0 (3.6)	112.7 (0.2)	111.9 (1.7)
18	Fipronil sulfone	1–50	y = 4.12 × 10^6^x + 2.51 × 10^5^	0.9996	1	2	118.0 (10.1)	108.7 (3.8)	117.0 (0.9)
19	Fipronil sulfoxide	1–50	y = 1.93 × 10^6^x + 1.75 × 10^5^	0.9988	1	2	116.6 (9.9)	95.9 (1.7)	117.1 (3.4)
20	Flubendiamide	1–50	y = 1.06 × 10^6^x − 1.82 × 10^5^	0.9978	1	2	91.5 (8.2)	96.3 (6.7)	101.8 (1.9)
21	Forchlorfenuron	1–50	y = 1.85 × 10^6^x − 4.60 × 10^5^	0.9993	1	2	112.5 (4.4)	115.0 (14.8)	84.0 (2.1)
22	Imidacloprid	1–50	y = 1.40 × 10^6^x + 5.42 × 10^4^	0.9978	1	2	100.5 (8.2)	108.4 (7.9)	109.9 (6.6)
23	ISAZOFOS	1–50	y = 5.99 × 10^6^x + 5.42 × 10^5^	0.9997	1	2	105.8 (13.6)	99.5 (5.3)	118.2 (1.3)
24	Isocarbophos	1–50	y = 6.23 × 10^5^x − 4.42 × 10^4^	0.9988	1	2	106.1 (5.2)	103.4 (10.2)	115.1 (1.6)
25	Malathion	1–50	y = 1.46 × 10^6^x − 3.01 × 10^4^	0.9989	1	2	109.7 (5.5)	116.0 (6.6)	117.5 (2.8)
26	Metalaxyl	1–50	y = 5.59 × 10^6^x + 8.35 × 10^5^	0.9997	1	2	118.6 (3.4)	117.9 (8.2)	104.2 (0.4)
27	Metronidazole	1–50	y = 4.54 × 10^5^x − 5.71 × 10^5^	0.9995	1	2	115.1 (4.2)	118.5 (12.1)	113.8 (9.1)
28	Phosfolan	1–50	y = 5.81 × 10^6^x + 3.86 × 10^5^	0.9994	1	2	100.0 (0.3)	117.7 (6.7)	112.5 (1.3)
29	Phosmet	1–50	y = 1.26 × 10^6^x + 1.78 × 10^5^	0.9985	1	2	106.1 (3.3)	89.4 (13.5)	104.1 (0.9)
30	Phoxim	1–50	y = 4.70 × 10^6^x + 3.46 × 10^6^	0.9999	1	2	70.0 (14.1)	110.9 (7.6)	117.4 (2.4)
31	Prochloraz	1–50	y = 2.04 × 10^6^x − 1.66 × 10^6^	0.9998	1	2	106.2 (2.9)	99.4 (1.3)	98.9 (0.1)
32	Prochloraz-desimidazole-amino	1–50	y = 1.98 × 10^6^x − 5.20 × 10^4^	0.9996	1	2	114.2 (19.1)	96.4 (6.5)	111.2 (3.2)
33	Profenofos	1–50	y = 1.94 × 10^6^x − 2.33 × 10^3^	0.9998	1	2	99.9 (6.8)	110.0 (17.6)	113.4 (1.7)
34	Propamocarb free base	1–50	y = 3.60 × 10^6^x − 8.19 × 10^4^	0.9971	1	2	108.0 (6.3)	115.2 (9.2)	111.8 (1.2)
35	Propiconazole	1–50	y = 1.97 × 10^6^x + 1.33 × 10^6^	0.9999	1	2	102.9 (0.6)	107.0 (6.9)	109.0 (2.1)
36	Proponit	1–50	y = 5.04 × 10^6^x + 4.19 × 10^5^	0.9992	1	2	116.4 (6.3)	110.5 (6.8)	102.5 (0.2)
37	Pyraclostrobin	1–50	y = 5.02 × 10^6^x + 1.19 × 10^5^	0.9994	1	2	114.2 (0.7)	86.5 (4.8)	104.5 (1.2)
38	Pyridaben	1–50	y = 2.71 × 10^6^x − 2.24 × 10^6^	0.9997	1	2	91.8 (8.9)	103.1 (6.3)	105.0 (2.4)
39	Pyrimethanil	1–50	y = 5.86 × 10^6^x + 3.70 × 10^5^	0.9998	1	2	90.4 (14.4)	72.0 (6.6)	90.1 (1.3)
40	S-metolachlor	1–50	y = 5.04 × 10^6^x + 4.19 × 10^5^	0.9992	1	2	116.4 (6.3)	110.5 (6.8)	112.5 (0.2)
41	Spinetoram J	1–50	y = 2.49 × 10^6^x − 4.40 × 10^4^	0.9990	1	2	108.4 (14.9)	97.0 (3.2)	118.6 (1.8)
42	Sulfadimethoxine	1–50	y = 1.11 × 10^5^x + 2.91 × 10^6^	0.9889	1	2	105.6 (11.5)	85.8 (6.9)	93.2 (1.3)
43	Sulfadimidine	1–50	y = 2.75 × 10^5^x + 5.0,8× 10^4^	0.9992	1	2	81.4 (3.1)	103.0 (2.7)	117.4 (2.0)
44	Sulfadoxine	1–50	y = 3.09 × 10^6^x − 2.03× 10^5^	0.9982	1	2	102.9 (9.1)	115.8 (1.2)	110.2 (5.7)
45	Sulfamerazine	1–50	y = 1.73 × 10^6^x − 4.06 × 10^3^	0.9985	1	2	117.3 (3.6)	115.7 (3.8)	103.4 (4.0)
46	Sulfameter	1–50	y = 2.62 × 10^6^x + 1.48 × 10^6^	0.9998	1	2	86.8 (13.3)	115.9 (1.3)	114.0 (4.5)
47	Sulfamonomethoxine	1–50	y = 9.00 × 10^4^x + 2.10 × 10^5^	0.9999	1	2	97.0 (1.5)	107.0 (3.3)	98.8 (0.5)
48	Sulfapyridine	1–50	y = 1.54 × 10^6^x + 1.90 × 10^6^	0.9996	1	2	74.4 (4.9)	112.8 (3.6)	116.3 (0.8)
49	Sulfaquinoxaline	1–50	y = 1.32 × 10^5^x + 1.29 × 10^4^	0.9994	1	2	94.5 (2.7)	102.3 (1.4)	93.1 (2.0)
50	Sulfisomidine	1–50	y = 3.67 × 10^6^x + 1.18 × 10^6^	0.9980	1	2	91.8 (7.7)	111.3 (4.6)	111.4 (0.5)
51	Tebuconazole	1–50	y = 2.23 × 10^6^x − 5.70 × 10^4^	0.9997	1	2	90.4 (18.8)	109.6 (6.7)	108.8 (0.1)
52	Triadimefon	1–50	y = 2.18 × 10^6^x − 3.30 × 10^5^	0.9998	1	2	104.6 (0.8)	119.1 (13.1)	111.0 (0.5)
53	Triazophos	1–50	y = 7.79 × 10^6^x − 1.96 × 10^6^	0.9998	1	2	81.4 (1.7)	113.9 (8.7)	107.8 (1.2)
54	Tricyclazole	1–50	y = 7.44 × 10^6^x − 3.43 × 10^6^	0.9998	1	2	117.5 (1.9)	80.6 (4.6)	82.1 (3.9)
55	Trimethoprim	1–50	y = 1.02 × 10^5^x + 1.46 × 10^5^	0.9997	1	2	91.9 (1.6)	114.7 (1.8)	116.4 (1.5)
56	Acephate	1–100	y = 1.33 × 10^5^x − 3.65 × 10^5^	0.9965	1	5	117.0 (2.4)	110.3 (18.5)	105.0 (6.7)
57	Acetamiprid	1–100	y = 3.38 × 10^6^x + 9.36 × 10^4^	0.9982	1	5	89.1 (10.8)	118.8 (3.0)	111.1 (5.5)
58	Ciprofloxacin	1–100	y = 5.39 × 10^4^x − 2.97 × 10^4^	0.9999	1	5	88.4 (6.1)	86.1 (2.5)	62.8 (3.5)
59	Cortisol	1–100	y = 2.13 × 10^5^x − 1.81 × 10^5^	0.9997	1	5	117.3 (13.4)	97.4 (1.5)	99.7 (3.9)
60	Danofloxacin	1–100	y = 3.03 × 10^5^x − 1.34 × 10^6^	0.9908	1	5	86.3 (11.5)	85.9 (9.8)	79.5 (8.2)
61	Difloxacin	1–100	y = 1.46 × 10^5^x + 5.75 × 10^5^	0.9983	1	5	88.0 (6.0)	97.0 (3.1)	104.2 (6.0)
62	Dimethoate	1–100	y = 1.24 × 10^6^x + 3.79 × 10^5^	0.9998	1	5	75.0 (0.5)	107.2 (12.8)	105.1 (3.9)
63	Dimetridazole	1–100	y = 4.65 × 10^5^x − 2.27 × 10^5^	0.9978	1	5	114.3 (4.9)	109.9 (8.3)	112.7 (2.9)
64	Enoxacin	1–100	y = 8.54 × 10^4^x − 1.33 × 10^6^	0.9915	1	5	62.7 (10.5)	68.4 (4.3)	62.9 (1.6)
65	Erythromycin	1–100	y = 3.18 × 10^3^x + 2.72 × 10^4^	0.9919	1	5	81.2 (5.4)	87.1 (4.6)	104.3 (0.9)
66	Enrofloxacin	1–100	y = 1.44 × 10^5^x + 1.62 × 10^5^	0.9996	1	5	97.9 (3.1)	70.2 (7.0)	88.8 (0.6)
67	Fenthion	1–100	y = 4.82 × 10^5^x + 2.06 × 10^5^	0.9992	1	5	83.4 (6.4)	91.6 (0.3)	109.8 (0.7)
68	Fenpropathrin	1–50	y = 4.82 × 10^5^x − 4.68 × 10^5^	0.9996	1	5	91.2 (1.9)	87.0 (3.9)	112.5 (2.7)
69	Fleroxacin	1–100	y = 1.57 × 10^5^x + 8.37 × 10^5^	0.9982	1	5	75.9 (6.4)	79.6 (0.2)	81.5 (3.4)
70	Flumequine	1–100	y = 7.85 × 10^4^x − 8.91 × 10^3^	0.9997	1	5	79.0 (9.1)	73.7 (3.6)	73.0 (1.1)
71	Lomefloxacin	1–100	y = 1.11 × 10^5^x − 1.28 × 10^5^	0.9997	1	5	84.0 (7.6)	89.1 (5.8)	100.4 (1.6)
72	Methylprednisolone	1–100	y = 2.42 × 10^5^x − 1.53 × 10^5^	0.9989	1	5	115.2 (5.3)	100.4 (5.2)	101.5 (0.2)
73	Norfloxacin	1–100	y = 1.03 × 10^5^x + 5.52 × 10^5^	0.9980	1	5	64.9 (5.7)	72.3 (3.8)	73.9 (4.5)
74	Ofloxacin	1–100	y = 1.61 × 10^5^x + 1.12 × 10^5^	0.9997	1	5	105.0 (12.1)	75.1 (5.4)	77.2 (4.7)
75	Oleandomycin	1–100	y = 2.03 × 10^6^x − 4.79 × 10^4^	0.9991	1	5	108.5 (1.0)	113.0 (0.1)	103.0 (0.8)
76	Omethoate	1–100	y = 2.22 × 10^6^x − 5.54 × 10^5^	0.9974	1	5	118.4 (7.9)	105.1 (15.0)	108.4 (0.2)
77	Orbifloxacin	1–100	y = 2.00 × 10^6^x − 1.71 × 10^3^	0.9996	1	5	87.8 (3.9)	89.4 (10.4)	88.6 (6.7)
78	Pefloxacin	1–100	y = 2.50 × 10^5^x − 2.95 × 10^6^	0.9944	1	5	79.3 (6.8)	65.3 (4.9)	62.0 (2.2)
79	Prednisone	1–100	y = 1.01 × 10^5^x − 5.44× 10^3^	0.9997	1	5	72.6 (6.2)	98.2 (9.2)	118.8 (2.8)
80	Roxithromycin	1–100	y = 6.74 × 10^4^x + 7.40 × 10^5^	0.9971	1	5	89.5 (7.1)	105.0 (5.7)	117.4 (0.7)
81	Sarafloxacin	1–100	y = 8.34 × 10^4^x + 3.36 × 10^5^	0.9920	1	5	61.7 (9.3)	68.4 (8.4)	63.1 (2.0)
82	Sparfloxacin	1–100	y = 1.37 × 10^4^x + 8.75 × 10^5^	0.9920	1	5	81.0 (3.8)	80.9 (4.2)	97.1 (2.7)
83	Sulfamethoxazole	1–100	y = 9.82 × 10^4^x + 2.59 × 10^5^	0.9986	1	5	104.0 (6.8)	115.9 (2.0)	117.4 (1.4)
84	Sulfafurazole	1–100	y = 1.39 × 10^6^x − 2.78 × 10^3^	0.9963	1	5	97.8 (3.1)	106.5 (2.5)	103.9 (1.6)
85	Thiamethoxam	1–100	y = 8.55 × 10^5^x + 1.83 × 10^5^	0.9998	1	5	117.8 (8.6)	118.7 (6.2)	117.7 (3.5)
86	Tilmicosin	1–100	y = 3.79 × 10^4^x − 8.01 × 10^2^	0.9977	1	5	114.3 (0.6)	104.9 (6.2)	118.1 (2.8)
87	Tylosin	1–100	y = 5.40 × 10^4^x + 4.29 × 10^5^	0.9970	1	5	102.0 (4.2)	94.2 (2.4)	114.1 (3.4)
88	Aldicarb	1–100	y = 1.73 × 10^5^x − 1.64 × 10^5^	0.9998	2	5	118.8 (6.3)	114.8 (11.4)	106.8 (2.0)
89	Azithromycin	1–100	y = 6.29 × 10^4^x + 7.50 × 10^5^	0.9972	2	5	100.5 (19.0)	100.6 (4.9)	118.8 (2.2)
90	Chlorbenzuron	1–100	y = 8.13 × 10^5^x − 2.62 × 10^5^	0.9998	2	5	83.4 (1.9)	78.7 (1.2)	79.0 (3.2)
91	Clothianidin	1–100	y = 5.58 × 10^6^x − 4.01 × 10^5^	0.9991	2	5	104.8 (13.4)	117.0 (0.2)	107.6 (5.7)
92	Fludrocortisone acetate	1–100	y = 1.82 × 10^5^x − 1.42 × 10^5^	0.9993	2	5	64.0 (5.3)	88.5 (8.2)	91.9 (4.0)
93	Hexaconazole	1–100	y = 1.26 × 10^6^x − 6.49 × 10^5^	0.9994	2	5	106.5 (13.4)	115.9 (13.5)	104.0 (1.3)
94	Pendimethalin	1–100	y = 4.39 × 10^5^x − 5.94 × 10^5^	0.9989	2	5	77.4 (3.7)	118.9 (8.4)	112.5 (2.3)
95	Prednisolone	1–100	y = 2.29 × 10^5^x − 1.62 × 10^5^	0.9996	2	5	106.2 (3.9)	96.6 (2.5)	101.2 (2.2)
96	Spiromesifen	1–100	y = 3.56 × 10^5^x − 2.29 × 10^5^	0.9995	2	5	109.6 (7.8)	104.4 (2.6)	104.0 (5.3)
97	Sulfabenzamide	1–100	y = 7.85 × 10^5^x + 5.97 × 10^5^	0.9997	2	5	93.8 (12.8)	116.3 (1.8)	114.2 (6.8)
98	Sulfacetamide	1–100	y = 1.87 × 10^3^x + 1.06 × 10^4^	0.9998	2	5	91.6 (8.8)	104.1 (17.7)	108.7 (4.1)
99	Sulfadiazine	1–100	y = 1.26 × 10^5^x − 4.62 × 10^4^	0.9999	2	5	90.0 (6.3)	101.6 (3.8)	107.5 (0.2)
100	Sulfathiazole	1–100	y = 1.02 × 10^5^x + 8.72 × 10^5^	0.9983	2	5	95.5 (6.7)	99.0 (3.5)	104.8 (7.8)
101	Sulfamethizole	1–100	y = 4.88 × 10^5^x + 1.81 × 10^4^	0.9990	2	5	95.2 (13.9)	115.0 (6.5)	117.3 (1.5)
102	Tebufenozide	1–100	y = 2.25 × 10^5^x − 8.14 × 10^5^	0.9975	2	5	87.8 (0.5)	101.3 (0.4)	94.3 (0.4)
103	Tetracycline	1–100	y =1.17 × 10^5^x – 9.01 × 10^4^	0.9999	2	5	102.8 (3.5)	67.5 (2.7)	71.3 (1.6)
104	Avermectin B1a	2–200	y = 1.23 × 10^5^x + 1.39 × 10^4^	0.9958	2	10	86.0 (8.9)	84.2 (6.4)	106.2 (3.7)
105	Chlorfluazuron	2–200	y = 3.73 × 10^5^x − 1.36 × 10^6^	0.9953	2	10	81.2 (10.8)	106.0 (0.6)	84.9 (1.3)
106	Deltamethrin	2–200	y = 2.71 × 10^4^x + 3.90 × 10^5^	0.9848	2	10	75.4 (17.3)	95.8 (16.3)	98.5 (7.6)
107	Demeton	2–200	y = 3.88 × 10^4^x − 7.48 × 10^3^	0.9870	2	10	95.1 (8.9)	110.3 (19.9)	121.6 (15.4)
108	Methomyl	2–200	y = 2.55 × 10^5^x − 5.70 × 10^5^	0.9986	2	10	91.1 (15.0)	116.8 (7.2)	110.4 (6.6)
109	Beclometasone	5–200	y = 1.71 × 10^4^x + 3.54 × 10^4^	0.9959	5	10	95.1 (12.1)	97.5 (8.7)	100.2 (10.4)
110	Chlortetracycline	5–200	y = 2.27 × 10^4^x + 2.91 × 10^4^	0.9996	5	10	70.0 (10.5)	77.1 (2.1)	73.7 (6.5)
111	Dimetridazole-2-hydroxy	5–200	y = 1.32 × 10^5^x + 5.25 × 10^4^	0.9968	5	10	81.6 (4.5)	87.3 (14.2)	118.0 (2.2)
112	Doxycycline (anhydrous)	5–200	y = 1.45 × 10^5^x − 2.36 × 10^5^	0.9999	5	10	61.8 (11.8)	79.4 (4.1)	75.5 (1.9)
113	Hydroxymetronidazole	5–200	y = 2.00 × 10^5^x − 1.24 × 10^4^	0.9902	5	10	79.3 (9.5)	105.3 (4.9)	109.9 (7.8)
114	Lufenuron	5–200	y = 2.15 × 10^4^x + 2.36 × 10^5^	0.9951	5	10	86.3 (12.8)	91.3 (1.9)	89.6 (4.4)
115	Oxadixyl	5–200	y = 2.07 × 10^6^x − 8.79 × 10^5^	0.9999	5	10	118.0 (12.6)	117.9 (11.2)	111.3 (1.1)
116	Oxytetracycline	5–200	y = 7.42 × 10^4^x + 6.40 × 10^4^	0.9999	5	10	63.6 (13.6)	70.8 (2.9)	64.2 (5.1)
117	Spiramycin	5–200	y = 2.44 × 10^4^x + 1.48 × 10^5^	0.9969	5	10	116.7 (1.9)	93.9 (5.8)	101.0 (2.0)
118	Sulfachlorpyridazine	5–200	y = 4.95 × 10^5^x + 2.18 × 10^5^	0.9984	5	10	84.7 (5.2)	99.6 (0.4)	113.0 (0.9)
119	Sulfaphenazole	5–200	y = 1.12 × 10^6^x − 1.43 × 10^5^	0.9982	5	10	78.0 (14.9)	113.6 (2.0)	114.5 (1.0)
120	Permethrin	5–200	y = 1.32 × 10^4^x + 4.75 × 10^5^	0.9994	5	10	76.1 (17.2)	98.8 (4.2)	104.4 (4.3)
121	Ronidazole	5–200	y = 1.18 × 10^5^x + 7.18 × 10^5^	0.9928	5	10	81.2 (10.5)	89.5 (6.4)	117.5 (1.8)

R^2^: determination coefficient, SDL: screening detection limit, LOQ: limits of quantitation quantitative, the values in parentheses refer to the RSD of recovery rates.

**Table 3:** Detection results of drug residues in real samples.

Classification	Targets	Screening Numbers	Quantitative Numbers	Mean Value (µg/kg)	Range (µg/kg)	Sample ID
Pesticides	Dimethomorph	21	14	211.37	2.13–1184	sample 22, 24, 26–31, 34–49
	Pyraclostrobin	13	12	8.90	2.01–24.4	sample 22, 24, 26–28, 34–40, 44
	Carbendazim	12	12	11.7	2.24–31.7	sample 21, 25–27, 29, 31, 36–40
	Metalaxyl	11	10	8.07	3.25–20.5	sample 22–23, 32–40
	Boscalid	10	9	24.2	3.03–48.2	sample 29, 31, 33, 35–40, 44
	Chlorantraniliprole	10	6	6.70	2.98–13.1	sample 2–10, 21–25
	Clothianidin	8	5	133.34	15.5–501.6	sample 22, 24–25, 27, 29, 37, 39–40
	Propamocarb-free base	7	6	184.6	2.49–299	sample 29, 31, 34–36, 38, 44
	Prochloraz-desimidazole-amino	6	6	11.0	2.84–16.0	sample 23, 25, 31, 37, 39–40
	Thiamethoxam	6	4	62.9	6.54–112.6	sample 22, 25, 32, 37, 39–40
	Difenoconazole	5	2	2.55	2.07–3.03	sample 34, 36–38, 40
	Imidacloprid	4	2	52.4	18.8–85.8	sample 2, 15–16, 24
	Pyridaben	3	3	21.9	2.97–35.7	sample 15, 21, 27
	Chlorbenzuron	2	1	9.95	9.95	sample 23, 25
	Dichlorvos	2	1	15.7	15.7	sample 39, 40
	Tebuconazole	2	2	35.5	18.2–52.7	sample 37, 40
	Azoxystrobin	2	0	—	—	sample 37, 39
	Acetamiprid	1	1	19.0	19.0	sample 31
	Chlorpyrifos	1	1	157	157	sample 31
	Prochloraz	1	1	12.6	12.6	sample 40
	Proponit	1	0	—	—	sample 28
Veterinary drugs	Doxycycline	3	0	—	—	sample 10, 26, 39
	Ofloxacin	1	0	—	—	sample 35

## Data Availability

Data will be made available on request.

## Supplementary Materials

The following supporting information can be downloaded at https://www.mdpi.com/article/10.3390/foods14193288/s1, Figure S1: Comparison of purification effects of different magnetic materials; Table S1: List of target compounds with their confirmation parameters; Table S2:The analyte numbers of different magnetic nanomaterials on ME.
