# Supplementary material for: Non-Targeted Screening and Quantitative Analysis of Pesticides and Veterinary Drug Residues in Brassica rapa chinensis Using an Improved Quechers Method Based on Magnetic Materials
Source: Foods. 2025 Sep 23;14(19):3288. doi: 10.3390/foods14193288 (PMC12523811; doi:10.3390/foods14193288)
Supplement: Supplementary file 1 [file foods-14-03288-s001.zip › foods-3835160-supplementary.pdf]

Supporting Information

Non-targeted screening and quantitative analysis of pesticide and veterinary drug residues in vegetables using an improved QuEChERS method based on magnetic materials

Minmin Tang[a], Yongbiao Ni[a], Tianli Zang[a], Wei Gao[a], Jinzhu Song[a], Jie Zou[a*], Danke Xu[b*]

[a] *Key Laboratory of Food Contact Materials Safety, State Administration for Market Regulation, Jiangsu Product Quality Testing & Inspection Institute, Nanjing, Jiangsu 210007, China.*

[b] *State Key Laboratory of Analytical Chemistry for Life Science, School of Chemistry and Chemical Engineering, Nanjing University, Nanjing 210023, China.*

1.1 Determination of co-extracts

The process were as follows: precisely aspirate 1 mL of crude extract (without magnetic bead purification) and 1 mL of purified solution by magnetic nanomaterial, then transfer them to glass tubes that have been dried and weighed at 110 ° C for 1 h. The solution in the glass tubes were blow to dry use a nitrogen evaporator at 60 ° C. Then, the glass tubes were placed in the oven again at 110 ° C for 1 h to remove moisture and reweigh. The amounts of co-extracts are the difference between the initial weight and final weight of the same tube.

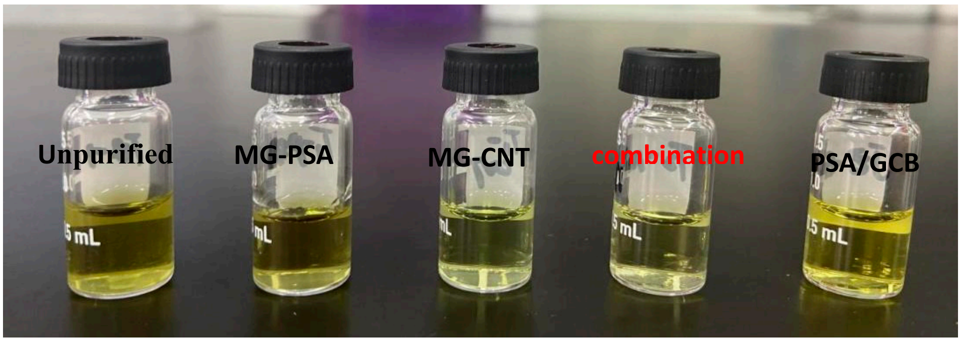

Figure S1. Comparison of purification effects of different magnetic materials.

Table S1. List of target compounds with their confirmation parameters.

No.	Compound Name	Formula	CAS	m/z	Mass error (ppm)	Addut	RT (min)	Fragment 1	Fragment 2	Fragment 3	Fragment 4	Fragment 5
1	Atrazine	$C_8H_{14}ClN_5$	1912-24-9	216.1010	-1.02	+H	4.87	174.05396	96.05553	132.03217	104.00087	79.00566
2	Acephate	$C_4H_{10}NO_3PS$	30560-19-1	184.0192	-1.66	+H	2.27	112.9996	49.01062	142.99246	N/S	N/S
3	Acetamiprid	$C_{10}H_{11}ClN_4$	135410-20-7	223.0745	-2.83	+H	2.85	126.01031	56.04937	90.03367	98.99944	N/S
4	Aldicarb	$C_7H_{14}N_2O_2S$	116-06-3	213.0668	-0.58	+Na	3.39	89.04185	116.05273	N/S	70.06505	N/S
5	Avermectin B1a	$C_{48}H_{72}O_{14}$	71751-41-2	890.5260	-0.44	$+NH_4$	12.90	305.21088	567.33148	307.22665	873.49829	N/S
6	Azithromycin	$C_{38}H_{72}N_2O_{12}$	83905-01-5	749.5158	0.66	+H	2.87	591.42163	116.10709	573.41113	158.11755	N/S
7	Azoxystrobin	$C_{22}H_{17}N_3O_5$	131860-33-8	404.1241	-1.17	+H	5.88	372.09756	344.10254	329.07925	172.03912	156.04425
8	Beclometasone	$C_{22}H_{29}ClO_5$	4419-39-0	453.1686	0.47	+HCOO	5.22	377.15244	407.16324	341.17563	N/S	N/S
9	Betamethasone	$C_{22}H_{29}FO_5$	378-44-9	437.1981	-0.23	+HCOO	5.05	361.18216	307.13416	345.15036	325.14487	292.11063
10	Boscalid	$C_{18}H_{12}Cl_2N_2O$	188425-85-6	343.0399	-0.60	+H	6.37	139.99001	112.03934	N/S	N/S	111.99477
11	Carbendazim	$C_9H_9N_3O_2$	10605-21-7	192.0768	-1.10	+H	2.50	160.05043	132.0556	105.04434	92.04943	65.03851
12	Carbofuran	$C_{12}H_{15}NO_3$	1563-66-2	222.1125	-1.23	+H	3.94	123.04392	165.09085	55.05416	91.05412	137.05949
13	Chlorantraniliprole	$C_{18}H_{14}BrCl_2N_5O_2$	500008-45-7	481.9781	-0.53	+H	5.26	283.92157	194.00029	177.0088	N/S	N/S
14	Chlorbenzuron	$C_{14}H_{10}Cl_2N_2O_2$	57160-47-1	309.0192	-0.84	+H	8.26	156.02094	138.99434	N/S	129.01007	N/S
15	Chlorfluazuron	$C_{20}H_9Cl_3F_5N_3O_3$	71422-67-8	539.9702	-1.74	+H	12.22	382.93597	141.01443	158.04095	186.9584	346.96011
16	Chlorpyrifos	$C_9H_{11}Cl_3NO_3PS$	2921-88-2	349.9336	-0.51	+H	10.95	197.92726	321.90204	293.87076	153.01321	124.98193
17	Chlortetracycline	$C_{22}H_{23}ClN_2O_8$	57-62-5	479.1216	-1.82	+H	2.42	462.09485	N/S	N/S	N/S	N/S
18	Ciprofloxacin	$C_{17}H_{18}FN_3O_3$	85721-33-1	332.1405	0.28	+H	2.44	288.14948	245.10829	N/S	N/S	N/S
19	Clarithromycin	$C_{38}H_{69}NO_{13}$	81103-11-9	748.4842	-1.03	+H	5.78	590.38959	158.11743	558.36353	N/S	N/S
20	Clothianidin	$C_6H_8ClN_5O_2S$	210880-92-5	250.0160	-0.10	+H	2.75	169.05412	131.96678	113.01685	110.07126	N/S
21	Cortisol	$C_{21}H_{30}O_5$	50-23-7	407.2075	0.12	+HCOO	4.32	331.19205	297.15009	315.16113	282.12646	125.06087
22	Danofloxacin	$C_{19}H_{20}FN_3O_3$	112398-08-0	358.1561	-2.02	+H	2.51	340.14545	N/S	N/S	N/S	N/S

No.	Compound Name	Formula	CAS	m/z	Mass error (ppm)	Addut	RT (min)	Fragment 1	Fragment 2	Fragment 3	Fragment 4	Fragment 5
23	Deltamethrin	$C_{22}H_{19}Br_2NO_3$	52918-63-5	503.9804	-1.05	+H	12.41	171.98801	278.90125	199.98314	170.98013	250.90636
24	Demeton	$C_8H_{19}O_3PS_2$	8065-48-3	259.0586	1.27	+H	4.17	127.01527	N/S	N/S	N/S	N/S
25	Dexamethasone	$C_{22}H_{29}FO_5$	50-02-2	437.1981	0.53	+HCOO	5.05	361.18237	307.13428	N/S	325.14474	N/S
26	Dichlorvos	$C_4H_7Cl_2O_4P$	62-73-7	220.9532	-0.46	+H	3.88	127.01539	N/S	144.98158	141.03113	78.99423
27	Difenoconazole	$C_{19}H_{17}Cl_2N_3O_3$	119446-68-3	406.0720	-0.32	+H	9.39	251.0024	188.03818	139.00595	337.03833	152.06207
28	Difloxacin	$C_{21}H_{19}F_2N_3O_3$	98106-17-3	400.1467	-0.44	+H	2.52	356.15649	N/S	299.09946	N/S	N/S
29	Diflubenzuron	$C_{14}H_9ClF_2N_2O_2$	35367-38-5	311.0393	-0.50	+H	7.88	141.0145	158.04105	N/S	140.03073	63.02292
30	Dimethoate	$C_5H_{12}NO_3PS_2$	60-51-5	230.0069	-1.41	+H	2.90	198.96451	88.02144	170.96964	142.99249	124.98183
31	Dimethomorph	$C_{21}H_{22}ClNO_4$	110488-70-5	388.131	0.16	+H	6.68	301.06259	165.05461	138.99458	70.02869	242.04953
32	Dimetridazole	$C_5H_7N_3O_2$	551-92-8	142.0611	-0.46	+H	2.49	96.0681	N/S	N/S	N/S	N/S
33	Dimetridazole-2-hydroxy	$C_5H_7N_3O_3$	936-05-0	158.0560	-0.90	+H	2.32	140.04539	55.04163	92.04943	N/S	N/S
34	Doxycycline (anhydrous)	$C_{22}H_{24}N_2O_8$	564-25-0	445.1605	1.19	+H	2.50	428.13412	N/S	N/S	N/S	N/S
35	Enoxacin	$C_{15}H_{17}FN_4O_3$	74011-58-8	321.1357	-0.21	+H	2.45	303.12396	N/S	N/S	N/S	N/S
36	Enrofloxacin	$C_{19}H_{22}FN_3O_3$	93106-60-6	360.1718	-1.06	+H	2.45	316.18185	245.10847	N/S	N/S	N/S
37	Erythromycin	$C_{37}H_{67}NO_{13}$	114-07-8	734.4685	-0.19	+H	5.35	576.37152	158.11732	N/S	N/S	N/S
38	Etofenprox	$C_{25}H_{28}O_3$	80844-07-1	394.2377	-2.71	$+NH_4$	5.35	177.12698	359.19992	305.15311	135.08015	183.07974
39	Fenpropathrin	$C_{22}H_{23}NO_3$	39515-41-8	350.1751	-0.67051	+H	11.71	125.09601	55.05417	57.06986	97.10114	69.06979
40	Fenthion	$C_{10}H_{15}O_4PS$	6552-13-2	263.0501	-1.27	+H	8.34	149.02295	205.08591	N/S	N/S	N/S
41	Fipronil	$C_{12}H_4Cl_2F_6N_4OS$	120068-37-3	434.9314	0.20	-H	8.05	183.01758	163.01132	249.95863	267.9895	182.00981
42	Fipronil desulfinyl	$C_{12}H_4Cl_2F_6N_4$	205650-65-3	386.9644	-0.30	-H	7.75	281.99265	350.98764	330.98196	324.98505	N/S
43	Fipronil sulfone	$C_{12}H_4Cl_2F_6N_4O_2S$	120068-36-2	450.9263	0.52	-H	8.75	414.94968	281.99268	243.98953	218.0097	82.96089

No.	Compound Name	Formula	CAS	m/z	Mass error (ppm)	Addut	RT (min)	Fragment 1	Fragment 2	Fragment 3	Fragment 4	Fragment 5
44	Fipronil sulfoxide	$C_{12}H_4Cl_2F_6N_4S$	120067-83-6	418.9365	0.25	-H	8.31	261.95859	57.97572	382.95993	313.96503	N/S
45	Fleroxacin	$C_{17}H_{18}F_3N_3O_3$	79660-72-3	370.1373	-0.17	+H	2.42	326.14801	269.08963	N/S	N/S	N/S
46	Flubendiamide	$C_{23}H_{22}F_7IN_2O_4S$	272451-65-7	681.016	0.73	-H	8.25	274.04739	254.04111	271.9216	405.96127	N/S
47	Fludrocortisone acetate	$C_{23}H_{31}FO_6$	514-36-3	467.2087	-0.57	+HCOO	5.14	421.20322	N/S	59.01387	N/S	N/S
48	Flumequine	$C_{14}H_{12}FNO_3$	42835-25-6	262.0874	0.36	+H	4.10	244.07649	N/S	N/S	N/S	N/S
49	Forchlorfenuron	$C_{12}H_{10}ClN_3O$	68157-60-8	248.0585	-1.12	+H	5.11	129.0213	111.0552	155.00056	93.04464	N/S
50	Hexaconazole	$C_{14}H_{17}Cl_2N_3O$	79983-71-4	314.0821	-0.05	+H	8.92	70.03992	158.97614	184.99202	N/S	N/S
51	Hydroxymetronidazole	$C_6H_9N_3O_4$	4812-40-2	188.0666	-1.74	+H	2.21	123.05518	144.04031	126.02973	N/S	N/S
52	Imidacloprid	$C_9H_{10}ClN_5O_2$	138261-41-3	256.0596	-1.47	+H	2.69	209.05869	175.09766	84.05552	212.05841	210.06665
53	ISAZOFOS	$C_9H_{17}ClN_3O_3PS$	42509-80-8	314.0490	-0.68	+H	6.94	162.04272	119.99577	114.96118	153.01317	243.97063
54	Isocarbophos	$C_{11}H_{16}NO_4PS$	24353-61-5	312.0430	-1.05	+Na	5.14	269.99619	236.00876	N/S	N/S	N/S
55	Lomefloxacin	$C_{17}H_{19}F_2N_3O_3$	98079-51-7	352.1467	2.02	+H	2.53	308.15598	265.11517	N/S	N/S	N/S
56	Lufenuron	$C_{17}H_8Cl_2F_8N_2O_3$	103055-07-8	510.9857	0.09	+H	10.97	158.04112	141.0146	327.97202	N/S	N/S
57	Malathion	$C_{10}H_{19}O_6PS_2$	121-75-5	331.0433	-0.75	+H	6.55	99.00749	127.03889	142.99252	N/S	78.99421
58	Metalaxyl	$C_{15}H_{21}NO_4$	57837-19-1	280.1543	-1.21	+H	5.03	220.13301	192.13812	160.11192	248.12793	148.11192
59	Methomyl	$C_5H_{10}N_2O_2S$	16752-77-5	163.0536	-1.29	+H	2.37	N/S	N/S	N/S	N/S	46.99501
60	Methylprednisolone	$C_{22}H_{30}O_5$	83-43-2	419.2075	-0.17	+HCOO	5.85	309.14993	343.19141	294.12628	N/S	N/S
61	Metronidazole	$C_6H_9N_3O_3$	443-48-1	172.0717	-1.06	+H	2.34	128.04518	N/S	N/S	N/S	N/S
62	Norfloxacin	$C_{16}H_{18}FN_3O_3$	70458-96-7	320.1405	-0.66	+H	2.48	276.15076	302.13022	233.10851	N/S	N/S
63	Ofloxacin	$C_{18}H_{20}FN_3O_4$	82419-36-1	362.1511	-2.94	+H	2.40	318.16119	261.1033	N/S	316.14539	N/S
64	Oleandomycin	$C_{35}H_{61}NO_{12}$	3922-90-5	688.4267	-1.08	+H	4.39	544.34747	158.11734	N/S	N/S	N/S
65	Omethoate	$C_5H_{12}NO_4PS$	1113-02-6	214.0297	-1.14	+H	2.33	142.99254	154.99251	182.9874	124.98205	61.01051

No.	Compound Name	Formula	CAS	m/z	Mass error (ppm)	Addut	RT (min)	Fragment 1	Fragment 2	Fragment 3	Fragment 4	Fragment 5
66	Orbifloxacin	$C_{19}H_{20}F_3N_3O_3$	113617-63-3	396.153	-0.61	+H	2.49	352.16306	N/S	N/S	N/S	N/S
67	Oxadixyl	$C_{14}H_{18}N_2O_4$	77732-09-3	279.1339	-0.83	+H	3.43	219.11256	102.05481	132.06535	192.10179	160.07579
68	Oxytetracycline	$C_{22}H_{24}N_2O_9$	79-57-2	461.1555	-1.39	+H	2.56	426.25	443.06	N/S	N/S	N/S
69	Pefloxacin	$C_{17}H_{20}FN_3O_3$	70458-92-3	334.1561	-0.43	+H	2.47	316.14578	N/S	N/S	N/S	N/S
70	Pendimethalin	$C_{13}H_{19}N_3O_4$	40487-42-1	282.1448	0.13	+H	11.05	212.06647	194.05571	118.05257	195.06374	71.0854
71	Permethrin	$C_{21}H_{20}Cl_2O_3$	52645-53-1	391.0862	-0.59	+H	13.25	183.08026	149.02322	113.05952	319.13269	167.03363
72	Phosfolan	$C_7H_{14}NO_3PS_2$	947-02-4	256.0225	-1.11	+H	2.60	167.98773	61.01056	127.01533	N/S	153.97214
73	Phosmet	$C_{11}H_{12}NO_4PS_2$	732-11-6	339.9838	-0.81	+Na	4.51	160.03926	133.02823	105.03352	95.04911	77.03847
74	Phoxim	$C_{12}H_{15}N_2O_3PS$	14816-18-3	299.0614	-0.85	+H	7.94	114.9612	N/S	242.99855	147.05516	163.0323
75	Prednisolone	$C_{21}H_{28}O_5$	50-24-8	405.1919	-0.42	+HCOO	4.20	329.17618	295.13406	N/S	280.11023	N/S
76	Prednisone	$C_{21}H_{26}O_5$	53-03-2	403.1762	-0.05	+HCOO	3.85	327.1604	357.16922	N/S	N/S	N/S
77	Prochloraz	$C_{15}H_{16}Cl_3N_3O_2$	67747-09-5	376.0381	-0.50	+H	8.30	N/S	70.02863	N/S	70.0651	N/S
78	Prochloraz-desimidazole-amino	$C_{12}H_{15}Cl_3N_2O_2$	139520-94-8	325.0272	-1.50	+H	8.88	282.02094	129.10199	86.09624	307.99988	72.0806
79	Profenofos	$C_{11}H_{15}BrClO_3PS$	41198-08-7	372.9424	-0.92	+H	10.13	302.86404	128.00221	114.96122	N/S	344.91104
80	Propamocarb free base	$C_9H_{20}N_2O_2$	24579-73-5	189.1598	-1.24	+H	2.37	102.05482	144.10175	74.02356	58.06505	N/S
81	Propiconazole	$C_{15}H_{17}Cl_2N_3O_2$	60207-90-1	342.0771	-2.33	+H	8.56	158.9762	69.06985	186.97078	N/S	204.98149
82	Proponit	$C_{15}H_{22}ClNO_2$	86763-47-5	284.1412	-0.66	+H	7.52	252.11488	176.14334	134.09645	133.08846	91.05431
83	Pyraclostrobin	$C_{19}H_{18}ClN_3O_4$	175013-18-0	388.1059	-0.22	+H	8.80	194.08126	163.06260	149.04710	133.05210	N/S
84	Pyridaben	$C_{19}H_{25}ClN_2OS$	96489-71-3	365.1449	-0.15	+H	12.30	147.11679	309.08237	132.09331	117.06981	119.08547
85	Pyrimethanil	$C_{12}H_{13}N_3$	53112-28-0	200.1182	-0.74	+H	4.99	82.06502	107.06026	183.09149	182.08359	80.04938
86	Ronidazole	$C_6H_8N_4O_4$	7681-76-7	201.0618	-1.15	+H	2.35	140.04527	N/S	N/S	N/S	N/S
87	Roxithromycin	$C_{41}H_{76}N_2O_{15}$	80214-83-1	837.5318	-2.47	+H	6.99	158.11745	679.43701	116.10696	116.07052	N/S

No.	Compound Name	Formula	CAS	m/z	Mass error (ppm)	Addut	RT (min)	Fragment 1	Fragment 2	Fragment 3	Fragment 4	Fragment 5
88	Sarafloxacin	$C_{20}H_{17}F_2N_3O_3$	98105-99-8	386.1311	-0.31	+H	2.55	342.14136	N/S	299.09885	N/S	N/S
89	S-metolachlor	$C_{15}H_{22}ClNO_2$	87392-12-9	284.1412	-0.66	+H	7.52	252.11488	176.14334	134.09645	133.08846	146.09642
90	Sparfloxacin	$C_{19}H_{22}F_2N_4O_3$	110871-86-8	393.1733	-0.91	+H	2.65	349.18454	292.1257	N/S	N/S	N/S
91	Spinetoram J	$C_{42}H_{69}NO_{10}$	187166-40-1	748.4994	-0.69	+H	9.80	142.12259	203.12762	98.09635	N/S	N/S
92	Spiramycin I	$C_{43}H_{74}N_2O_{14}$	8025-81-8	843.5213	-1.42	+H	3.03	174.11226	N/S	N/S	N/S	N/S
93	Spiromesifen	$C_{23}H_{30}O_4$	283594-90-1	371.2217	-0.85	+H	11.34	73.04672	N/S	285.00946	N/S	91.05722
94	Sulfabenzamide	$C_{21}H_{24}Cl_2O_4$	127-71-9	277.0641	-1.29	+H	2.75	156.01117	N/S	N/S	N/S	N/S
95	Sulfacetamide	$C_8H_{10}N_2O_3S$	144-80-9	215.0485	-0.98	+H	2.23	156.0112	92.04932	108.04422	N/S	N/S
96	Sulfachlorpyridazine	$C_{10}H_9ClN_4O_2S$	80-32-0	285.0208	-0.42	+H	2.60	156.01129	N/S	N/S	N/S	N/S
97	Sulfadiazine	$C_{10}H_{10}N_4O_2S$	68-35-9	251.0597	-1.57	+H	2.32	156.01131	N/S	N/S	N/S	N/S
98	Sulfadimethoxine	$C_{12}H_{14}N_4O_4S$	122-11-2	311.0809	-0.72	+H	2.65	156.01122	156.07661	108.04425	N/S	N/S
99	Sulfadimidine	$C_{12}H_{14}N_4O_2S$	57-68-1	279.0910	-1.59	+H	2.48	204.04393	124.08682	156.01131	N/S	N/S
100	Sulfadoxine	$C_{12}H_{14}N_4O_4S$	2447-57-6	311.0809	-0.72	+H	3.60	156.01122	156.07661	108.04425	N/S	N/S
101	Sulfafurazole	$C_{11}H_{13}N_3O_3S$	127-69-5	268.075	-0.22	+H	2.63	156.01132	113.07085	N/S	N/S	N/S
102	Sulfamerazine	$C_{11}H_{12}N_4O_2S$	127-79-7	265.0754	-0.66	+H	2.42	156.01134	172.01718	190.02806	N/S	N/S
103	Sulfameter	$C_{11}H_{12}N_4O_3S$	651-06-9	281.0703	-0.79	+H	2.49	156.01118	215.09201	N/S	N/S	N/S
104	Sulfamethizole	$C_9H_{10}N_4O_2S_2$	144-82-1	271.0318	-0.30	+H	2.46	156.01137	108.0443	N/S	N/S	N/S
105	Sulfamethoxazole	$C_{10}H_{11}N_3O_3S$	723-46-6	254.0594	-0.37	+H	2.59	156.01128	N/S	N/S	N/S	N/S
106	Sulfamonomethoxine	$C_{11}H_{12}N_4O_3S$	1220-83-3	281.0703	-0.79	+H	2.49	156.01118	215.09201	N/S	N/S	N/S
107	Sulfaphenazole	$C_{15}H_{14}N_4O_2S$	526-08-9	315.091	-0.34	+H	2.80	156.01137	N/S	N/S	N/S	N/S
108	Sulfapyridine	$C_{11}H_{11}N_3O_2S$	144-83-2	250.0645	-0.74	+H	2.38	156.01134	184.08702	95.06036	N/S	N/S
109	Sulfaquinoxaline	$C_{14}H_{12}N_4O_2S$	59-40-5	301.0754	-0.78	+H	3.07	156.01128	108.04435	92.04942	146.07117	N/S
110	Sulfathiazole	$C_9H_9N_3O_2S_2$	72-14-0	256.0209	-0.78	+H	2.32	156.01122	108.04424	92.04932	N/S	N/S

No.	Compound Name	Formula	CAS	m/z	Mass error (ppm)	Addut	RT (min)	Fragment 1	Fragment 2	Fragment 3	Fragment 4	Fragment 5
111	Sulfisomidine	$C_{12}H_{14}N_4O_2S$	515-64-0	279.0910	-1.59	+H	2.48	204.04393	124.08682	156.01131	N/S	N/S
112	Tebuconazole	$C_{16}H_{22}ClN_3O$	107534-96-3	308.1524	-0.45	+H	8.42	70.03992	N/S	125.01517	151.03114	57.06988
113	Tebufenozide	$C_{22}H_{28}N_2O_2$	112410-23-8	353.2224	-0.77	+H	7.94	N/S	79.05423	N/S	133.065	N/S
114	Tetracycline	$C_{22}H_{24}N_2O_8$	60-54-8	445.1605	1.19	+H	2.41	410.12378	427.14987	428.13412	N/S	N/S
115	Thiamethoxam	$C_8H_{10}ClN_5O_3S$	153719-23-4	292.0266	-1.08	+H	2.54	211.06461	131.96677	N/S	210.05681	N/S
116	Tilmicosin	$C_{46}H_{80}N_2O_{13}$	108050-54-0	869.5733	-0.65	+H	3.60	N/S	174.1124	N/S	N/S	N/S
117	Triadimefon	$C_{14}H_{16}ClN_3O_2$	43121-43-3	294.1004	-0.44	+H	6.72	70.03989	197.07265	N/S	N/S	69.0698
118	Triazophos	$C_{12}H_{16}N_3O_3PS$	24017-47-8	314.0723	-0.92	+H	6.97	162.06602	114.96117	119.06023	N/S	92.04932
119	Tricyclazole	$C_9H_7N_3S$	41814-78-2	190.0433	-0.99	+H	3.18	136.02136	109.01048	163.03226	92.04932	65.03847
120	Trimethoprim	$C_{14}H_{18}N_4O_3$	738-70-5	291.1452	-0.73	+H	2.39	230.11597	123.06633	261.09802	275.11371	N/S
121	Tylosin	$C_{46}H_{77}NO_{17}$	1401-69-0	916.5264	-0.73	+H	5.24	174.11267	772.44757	N/S	N/S	N/S
